# Is emotional intelligence linked with academic achievement? The first TEIQue-SF study in a sample of Saudi medical rehabilitation students

**DOI:** 10.1016/j.amsu.2022.103726

**Published:** 2022-05-12

**Authors:** Lujain Abu Alkhayr, Roaa Alshaikh, Layan Alghamdi, Alaa Alshaikh, Fahad Somaa, Faraz Ahmed Bokhari

**Affiliations:** a Occupational Therapy Department, Faculty of Medical Rehabilitation Sciences, King AbdulAziz UniversityJeddah, Saudi Arabia; b Department of Physiology, Shaikh Zayed Federal FPGMI, Lahore, Pakistan

**Keywords:** Trait emotional intelligence, TEIQue-SF, Academic performance, Medical rehabilitation

## Abstract

**Objective:**

The present study examined the relationship of the Trait Emotional Intelligence Questionnaire Short Form (TEIQue-SF) and academic achievement (GPA). Analyses were performed using a sample of Saudi-origin medical rehabilitation undergraduate students (*N* = 130). The present study examined the psychometric properties of the Chinese version of the Trait Emotional Intelligence Questionnaire Short Form (TEIQue-SF). Analyses were performed using a sample of undergraduates (*N* = 585) recruited from four universities across China.

**Methods:**

One hundred thirty medical rehabilitation students completed the Trait Emotional Intelligence Questionnaire-Short Form (TEIQue-SF). Descriptive and inferential statistical analyses were carried out to elucidate relationships (or the lack of the same) between various variables.

**Results:**

Whole sample alpha coefficient value for global trait EI was 0.84, while the same for trait EI factors ranged from 0.51 to 0.76. Global Trait EI was found higher in males than in females (Female students median score: 17 ± 2.56 VS Male students median score: 18 ± 3.67; U: 1667, p 0.04). A positive and statistically significant relationship was found between Well-being and the three other factors (with Self-control [r(128), 0.413, p 0.01]; with Emotionality [r(128), 0.518, p 0.01], with Sociability [r(128), 0.490, p 0.01]). Sociability was found to have a similar positive relationship with Self-control [r(128), 0.239, p 0.05] and Emotionality [r(128), 0.490, p 0.01] respectively. Furthermore, GPA was found to have a negative (not statistically significant) relation with Sociability. Overall, there was no association found between trait EI and GPA.

**Conclusions:**

The present study is one of two studies that has investigated the train EI-academic achievement link in healthcare-related students. Our findings resonate with existing literature on the subject.

## Highlights

• The present study aims to use TEIQue-SF (shorter version of the long form TEIQue instrument) to study trait EI measures on a sample of undergraduate medical rehabilitation students in a Saudi public sector university.
• The choice of the shorter version is based on conciseness, predictive validity, and good fundamental psychometric properties covering student and nonstudent samples across the world.
• There is a considerable amount of interest around emotional IQ and how it affects us. TEI in this regard has come up with some important theorizations, one of them being its link with academic achievement.
• We conclude that the TEi construct may be diverse, and might have more indirect pathways that link it to academic performance.

## 1 Background

Emotional intelligence (EI) enables people to recognize their emotions and use them to refine their decision-making [[1], [2], [3], [4], [5]]. It has been discovered that distinguishing between emotions can help guide people's actions and improve their problem-solving abilities [5]. EI has been recognized as a key component in the success of various healthcare professionals [1].

Since its introduction into the research literature, emotional intelligence (EI) has piqued the interest of many researchers [6]. Generally speaking, EI attempts to explain unique emotion-based variability amongst different individuals [7,8]. Trait EI is an often-cited depiction of EI, which characterizes it as a group of behavioural dispositions and self-perceptions related to one's emotions that are inherently found underlying the personality hierarchies [[9], [10], [11], [12]]. Trait EI is assessed by employing self-report instruments [9], and many such tools are available [13,14], one of which is called the Trait Emotional Intelligence Questionnaire – TEIQue [15]. Literature reveals that studies employing TEIQue in undergraduate student samples have found many positive associations of global trait EI with: mental resilience [16], greater use of coping mechanisms [17], and better academic performance [8,18]. Most of these studies have been done in Western countries.

The link between train EI and academic performance is an important and interesting one. It is known that only academic knowledge is not sufficient to achieve success as a healthcare student or in real-life practice [2,5]. Higher EI has been found associated with higher critical thinking and reasoning skills, better self-management, improved patient interaction, academic success, and lesser stress among health sciences students [[19], [20], [21], [22], [23]]. All of this leads to better comprehensive patient care [24]. Importantly, EI skills are learnt and/or improved [25].

As noted above, trait EI was been mainly developed and studied in Western contexts, and hence requires an appraisal in non-Western perspectives [26]. A limited number of studies have been conducted on emotional intelligence and its effect on academic performance in Saudi Arabia. Some studies conducted were conducted on Saudi undergraduate English language students to determine the relationship between EI and academic achievement [27,28]. Other studies with similar objectives used business [29] and medical students [30] as samples to study the EI link with academic attainment. TEIQue was not used in any of these studies.

The present study aims to use TEIQue-SF (shorter version of the long form TEIQue instrument) [15] to study trait EI measures on a sample of undergraduate medical rehabilitation students in a Saudi public sector university. The choice of the shorter version is based on conciseness, predictive validity, and good fundamental psychometric properties covering student and nonstudent samples across the world [[31], [32], [33], [34], [35], [36], [37]].

## 2 Materials and methods

### 2.1 Participants

The participants in this cross-sectional study were Saudi undergraduate medical rehabilitation students, enrolled in King Abdulaziz University, Jeddah, KSA. The participants (*N* = 130, 73 men, 57 women) were recruited from all five constituent departments of the Faculty of Medical Rehabilitation Sciences. Their ages ranged from 20 to 25 years (*M* = 20.87, *SD* = 1.01).

This Study was approved by King Abdulaziz University of Medical Rehabilitation Sciences' ethics committee with reference letter number of: FMRS-EC2021-01. After informed consent was obtained, medical rehabilitation students from Abdulaziz University in Jeddah, Saudi Arabia, during the education year 2020/2021 were enrolled in the study. Students from all the years of study were included.

Our methodology is also in line with STROCSS criteria [38].

The research is registered at research registery. The Unique Identifying Number UIN from http://www.researchregistry.com*: researchregistry7840*.

### 2.2 Instrument

We utilized the Trait Emotional Intelligence Questionnaire-Short Form - TEIQue-SF [15]. It consists of 30-item measure that evaluates global trait EI and four trait EI factors: Well-Being, Self-Control, Emotionality, and Sociability. All of these were calculated in accordance with the TEIQue-SF scoring key, obtained from Petrides' university laboratory website i.e., global trait EI was calculated by adding up items 3, 14, 18, and 29 (since only they are said to contribute to global trait EI) and the four factors were calculated by following the precise directions given on the author's website mentioned above. TEIQue-SF uses a 7-point Likert scale ranging from 1:*completely disagree* or *strongly disagree* through to 7: *completely agree* or *strongly agree*. Thequestionnaire was first uploaded on Google Forms, and then the link of the form was sent electronically to all the students enrolled in the program through WhatsApp. Informed consent was obtained from all the participating students.

Academic performance was measured by the participants' GPA. The university has a grading system with 0 being the minimum GPA and 5 being the highest. Students with a GPA of 2.75 are considered failed, 2.75–3.75 as good, 3.75–4.50 as very good, and 4.5 upwards as excellent in academics.

### 2.3 Data analysis

The goal of the present study was twofold. Firstly, to determine the reliability of the *TEIQue-SF*. Secondly, the descriptive and inferential evaluation of global trait EI and the four trait EI factors (well-being, self-control, emotionality, and sociability) across gender and GPA. Descriptive variables were expressed as means and percentages. Normally distributed data was analyzed using student *t*-test, while non-normally distributed data was evaluated by Mann Whitney *U* test. Correlation was performed using Pearson correlation coefficient. SPSS version 21 (IBM) and MS Excel 2016 (Microsoft) were used to analyze data.

## 3 Results

### 3.1 Normality testing

Since our data size was modest, we used Shapiro-Wilk for normality testing. Out of 4 EIQ parameters ‘Wellbeing’ and ‘Emotionality’ were found not normally distributed (p 0.002 for each), while ‘Self-control’ and ‘Sociability’ were found to be normally distributed (p 0.095 and 0.086 respectively). It is worth mentioning that the non-normal distribution of the ‘Well-Being’ and perhaps ‘Emotionality’ indicates a component of construct validity and not weakness of data. Research shows that people tend to rather happy than neutral. Only in the very poor countries does the number of Unhappy individuals equal happy ones in the poorest of countries only [39].

### 3.2 Descriptive statistics

Our sample comprised of 130 participants out of whom 57 (44%) were females and 73 (56%) were males. The mean age of the sample was 20.87 years (SD 1.01). All participants were of Saudi origin. Most of the participants were unmarried (92%). Also majority of them lived in urban areas (90%). Mean GPA was found to be 4.21 (SD 0.33). Table 1 details descriptive data, and Cronbach alphas of the TEIQue factors and global trait EI. Gender-stratified scores of trait EI factors and global trait EI divided into three categories: 1–3, 3.1–5 and 5.1–7 **(**Table 2**)**. Most of the participants scored between 3.1 and 5 for all factors (except Well-being) and global trait EI.

**Table 1:** TEIQue factor means, standard deviations, and internal consistencies.

n = 130	Mean	Std. Deviation	Minimum	Maximum	α
Well Being	5.05	1.09	2.33	7.00	0.76
Self-Control	4.21	0.91	2.17	6.67	0.51
Emotionality	4.49	0.84	2.25	6.38	0.51
Sociability	4.50	0.94	2.17	6.67	0.60
Global Trait EI	4.59	0.82	3.25	6.50	0.84

**Table 2:** TEIQue Factor Scores by Score Range (stratified by gender).

Factor/Score Range	Female Student Scores	Male Student Scores
	n, %age	n, %age
Well-being		
Score: 1-3	2, 3.5	0, 0
Score: 3.1–5	18, 31.6	39, 53.4
Score 5.1–7	37, 64.9	34, 46.6
Self-control		
Score: 1-3	9, 15.8	7, 9.6
Score: 3.1–5	37, 64.9	52, 71.2
Score 5.1–7	11, 19.3	14, 19.2
Emotionality		
Score: 1-3	5, 8.8	0, 0
Score: 3.1–5	37, 64.9	59.3, 74
Score 5.1–7	15, 26.3	19, 26
Sociability		
Score: 1-3	6, 10.5	0, 0
Score: 3.1–5	42, 73.7	50, 68.5
Score 5.1–7	9, 15.8	23, 31.5
Global Trait EI		
Score: 1-3	3, 5.3	0, 0
Score: 3.1–5	50, 87.7	50, 68.5
Score 5.1–7	4, 7.0	23, 31.5

### 3.3 Reliability analysis

Across the whole sample, the alpha coefficient value for global trait EI was large (α = 0.84). Values for the trait EI factors ranged from small (α = 0.51) to large (α = 0.76). These values are similar to other published studies [40].

### 3.4 Inferential statistics

Mann-Whitney *U* test was used to study differences for well-being and emotionality between genders, since this data was not normal distributed. However, self-control and sociability variables were normally distributed and were tested with student t-test. Global Trait EI was found higher in males than in females (Female students median score: 17 ± 2.56 VS Male students median score: 18 ± 3.67; U: 1667, p 0.04). None of the other results reached statistical significance **(**Table 3, Table 4**).**

**Table 3:** Mann-Whitney U testing of Well-Being and Emotionality Variables (stratified by Gender).

Variable	Well-Being	Emotionality	Global Trait EI
*n* = Female:57, Male:73			
Females			
Median	5.33	4.63	17.00
Std. Deviation	1.14	0.92	2.56
Males			
Median	5.00	4.63	18.00
Std. Deviation	1.04	0.78	3.67
Mann-Whitney U	1755	2021	1664
P	0.12	0.78	0.04*

*p < 0.05.

**Table 4:** Student *t*-Test of Self-Control and Sociability Variables (stratified by Gender).

Variable		
*n* = Female:57, Male:73	Self-Control	Sociability
Females		
Mean	4.21	4.36
Std. Deviation	0.96	1.03
Males		
Mean	4.21	4.61
Std. Deviation	0.88	0.86
P	0.94	0.14

### 3.5 Correlation analysis

**Fig. 1:**
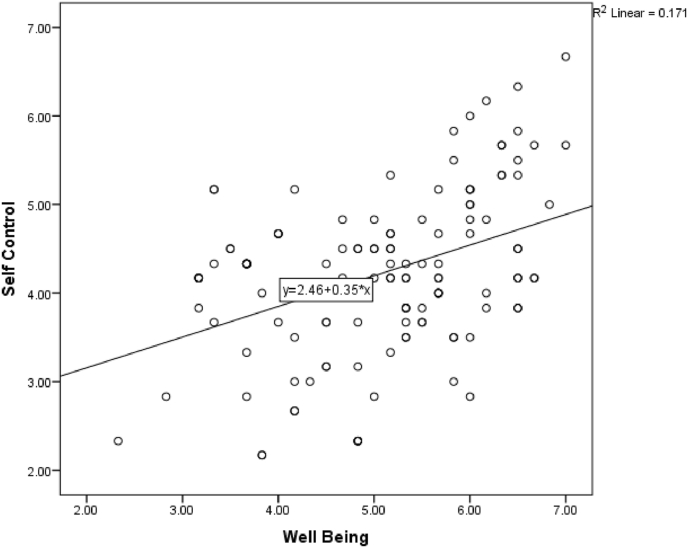
Scatter plot diagram showing positive relationship between Well-being and Self-control [r(128), 0.413, p 0.01].

A positive and statistically significant relationship was found between Well-being and the three other factors (with Self-control [r(128), 0.413, p 0.01]; (Fig. 1), with Emotionality [r(128), 0.518, p 0.01], with Sociability [r(128), 0.490, p 0.01]). Furthermore, Sociability was found to have a similar positive relationship with Self-control [r(128), 0.239, p 0.05] and Emotionality [r(128), 0.490, p 0.01] respectively. GPA was found to have a negative relation with Sociability, though it was not statistically significant.

## 4 Discussion

We set out two objectives for this study namely, to determine the reliability of the *TEIQue-SF* in a sample of Saudi medical rehabilitation students, and secondly, to study sought to determine any associations between students’ EI scores and academic achievement (GPA). Our study is unusual in that it is the only one that we are aware of that looked at the relationship between TEIQue-SF and academic achievement in medical rehabilitation students. In fact we could only find one study on the subject in the healthcare sector (with nursing students) till date; this study looked at 81 nursing students from a university in Sydney and their responses to a modified Emotional intelligence questionnaire. The results showed a significant correlation between EI scores and critical thinking (p < 0.001), help-seeking (p < 0.003), and peer learning (p < 0.004) but not for extrinsic goal orientation. Overall EI was a significant predictor of academic achievement (P < 0.023) [41].

Reliability of internal consistency, as calculated by Cronbach alpha, came out to be 0.76 for well-being, 0.51 for self-control, 0.51 for emotionality, 0.60 for sociability, and 0.84 for Global trait EI. Findings were satisfactory and comparable with similar other studies **(**Table 5**).**

**Table 5:** Reliability score comparison.

Variable	Mean, SD	Α
Current study		
Global trait EI	4.73, 0.69	0.84
Well-being	5.17, 1.04	0.76
Self-control	4.19, 0.91	0.51
Emotionality	4.79, 0.85	0.51
Sociability	4.77, 0.87	0.60
*Chinese Sample*		
Global trait EI	4.73, 0.64	0.88
Well-being	5.10, 0.96	0.82
Self-control	4.53, 0.80	0.65
Emotionality	4.87, 0.74	0.65
Sociability	4.32, 0.68	0.47
*Canadian Sample*		
Global trait EI	4.73, 0.69	0.88
Well-being	5.17, 1.04	0.85
Self-control	4.19, 0.91	0.67
Emotionality	4.79, 0.85	0.67
Sociability	4.77, 0.87	0.71
Turkish study		
Global trait EI	n/a	0.81
Well-being	n/a	0.72
Self-control	n/a	0.70
Emotionality	n/a	0.66
Sociability	n/a	0.70

Gender differences for EI revealed an interesting result. Global Trait EI was found higher in males than in females (Female students median score: 17 ± 2.56 VS Male students median score: 18 ± 3.67; U: 1667, p 0.04), negating the popular psychology perception of ‘‘IQ is male and EQ is female’‘. Moreover, we found male and female scores to be very close for all the four EI factors (Table 3, Table 4). And the standard deviations are also comparable in for all factors, demonstrating similar dispersions in the responses of both genders. These results are similar to those found by another study in 2009, which looked at 1721 individuals (912 females, 764 males, and 61 unreported) with a mean age of 29.65 (SD = 11.94) [15].

As mentioned earlier, most of the participants’ EI scores were found to be within the 3.1–5 (medium) range for all factors (except Well-being), and global trait EI for both genders. For Well-being, most females (n = 37, 64.9 %age) scored in the 5.1–7 (higher) range, while most males (n = 39, 53.4 %age) scored in the 3.1–5 (medium) range. High Well-being scores indicate a sense of well-being, stemming from past attainments, including future expectations. High-achievers on this scale generally are positive-minded, happy, and fulfilled. A medium score on the Self-control factor indicates that you have some control over your urges and desires. This factor is also a measure of the ability to regulate external pressures and stress. Low self-control results in inflexibility. Scores in Emotionality reflect the presence of a reasonable range of emotion-related skills. Perception and expression of emotions, along with the ability to refine and sustain close relationships with others comes naturally to such people. Lastly, the sociability factor underlines the importance of social relationships and influence. Our sample scores on this factor indicates that our students are reasonably good in their social interaction, based on perceived good listening and communication skills [42].

We did not find any significant association between TEI parameters and GPA. The evidence regarding the relationship between TEI and academic achievement is not straightforward. While there are a number of studies that found a positive, statistically significant association between the two [18,41,[43], [44], [45], [46]], others failed to do so [45,47], while one study found mixed patterns [48]. In one study that failed to find a relationship, out of the 193 undergraduate students, those who were in a mid-range GPA had a higher “well-being” factor score than students in a higher and lower range GPA. Otherwise, the results did not show any significant relationship between Global trait EI and academic scores [46]. In the study with mixed results, a trait emotional intelligence questionnaire-child form (TWIQue-CF) was distributed to 565 children in primary schools, of which 274 were boys and 286 were girls, and the mean age was 9.12 years (SD = 1.27 years). The results showed a humble association between trait EI and academic achievement restricted to students in year 3 only. Moreover, those students with special education needs scored lower on trait EI compared to those who did not have special education needs [47].

It is tempting to assume that TEI would (or should) facilitate academic achievement, since students with higher TEI scores possess a better ability to regulate their emotions, extend the range of methods for engaging in academic tasks and enrich/maintain attention to academic activities. In theory, all this makes sense. However, there are aspects of the TEI, like the Sociability component, that need a closer look. Sociability in the TEI context relates to having a pro-social disposition. This tendency towards social activity may in fact hinder students to fully focus on academic work [49,50]. This might explain our finding of GPA being in a negative relationship with Sociability (though not statistically significant).Related to this, researchers reported a similar effect of sociability component of TEI having no contribution towards academic achievement [51,52]. In one of these studies, 874 students (433 boys & 441 girls) aged 15–16 years from 24 British schools were given the trait emotional intelligence questionnaire to complete. The results revealed that all aspects of the sociability factor, including social awareness, emotion management, and assertiveness were not found to be significantly associated with academic scores. In conclusion, being adept at social interaction and communicating efficiently did not have an impact on the student's academic performance [50].Another component of the TEI is tendencies towards the management of others' emotions, which may serve as a social stressor and interfere with academic activities. Hence whether the whole construct of EQ, as we know it today, is good for students is still a question [12]. And therefore, the TEI-achievement link too needs more reconsideration with an open mind to include the possibility that there may be various, divergent ways that TEI affects student performance in exams.

A pertinent observation was made by Perera and DiGiacomo (2013) [53] in their meta-analysis (comprising of data from 47 independent samples and over 8700 participants). While they found a near-moderate association of global TEI with achievement (r = .20, 95% CI = .16–0.24), the said association was moderated by age and academic level of the participants. That is, the effect increased as the age decreased, and it was stronger in primary samples than tertiary ones. Hence it may be the case that the interpersonal processes that supposedly link TEI with academic achievement may be prominent in the early educational years where learning is usually more collaborative. Lastly, could it be that the association between TEI and achievement may be mainly indirect and not via direct pathways? [54] conducted a meta-analysis, by extracting 74 effect sizes from 48 independent samples (total sample size = 10,292). While examining the pathways that link TEI with achievement, it was found that higher TEI was in fact linked with better academic attainment *indirectly* (by employing coping strategies and academic engagement), and not *directly*. This was represented in the results as a moderate validity coefficient for trait EI (r = 0.20, 95% CI), with homogeneity analysis showing heterogeneity in the effects, and tests of moderation showing that the summary effect increased as a function of academic level. To conclude, the effect/s of TEI on secondary and higher education seems to be more complex and divergent than what the current body of evidence espouses.

### 4.1 Study limitations

Our sample size was modest. Also both genders should have been equally, or near equally, represented. There is also the issue of generalizability, since only one type of students were recruited, and from the same institution.

## 5 Conclusions and future directions

There is a considerable amount of interest around emotional IQ and how it affects us. TEI in this regard has come up with some important theorizations, one of them being its link with academic achievement. There are however gaps in theoretical knowledge linking these two constructs lack of conceptual clarity along with a dearth of the number of pertinent studies on the subject, especially in the healthcare field, poses a unique challenge. Our study contributes to the fundamental debate concerning the causal relationship between train EI and academic achievement, or the lack of. We conclude that the TEi construct may be diverse, and might have more indirect pathways that link it to academic performance.

## Ethical approval

This Study was approved by King Abdulaziz University of Medical Rehabilitation Sciences' ethics committee with reference letter number of: FMRS-EC2021-01.

## Funding source

None.

## Author contribution

Lujain Abu Alkhayr: study design, data collection, data interpretation, writing paper. RoaaAlshaikh: study design, data collection, data interpretation, writing paper. Layan Alghamdi: study design, data collection, data interpretation, writing paper. AlaaAlshaikh: study design, data collection, data interpretation, writing paper. Fahad Somaa: study concept, study design, data interpretation, writing paper. Faraz Ahmed Bokhari: data interpretation, writing paper.

## Consent

Informed consent was taken from all students.

## Guarantor

The Guarantor is the one or more people who accept full responsibility for the work and/or the conduct of the study, had access to the data, and controlled the decision to publish1. Lujain Abu Alkhayr.2. RoaaAlshaikh3. Layan Alghamdi4. AlaaAlshaikh5. Fahad Somaa|6. Faraz Ahmed Bokhari.

## 6 The references are provenance and peer review

Not commissioned, externally peer-reviewed.

## Declaration of competing interest

None.
